# Transcriptional Regulation of GDF15 by EGR1 Promotes Head and Neck Cancer Progression through a Positive Feedback Loop

**DOI:** 10.3390/ijms222011151

**Published:** 2021-10-15

**Authors:** Yanli Jin, Seung-Nam Jung, Mi Ae Lim, Chan Oh, Yudan Piao, Hae Jong Kim, Lihua Liu, Yea Eun Kang, Jae Won Chang, Ho-Ryun Won, Kunho Song, Bon Seok Koo

**Affiliations:** 1 Department of Medical Science, Chungnam National University College of Medicine, Daejeon 35015, Korea; jinyanli5678@gmail.com (Y.J.); ohchanny@naver.com (C.O.); yudan0329@gmail.com (Y.P.); thename45@gmail.com (H.J.K.); xjlh0305@naver.com (L.L.); strive1005@hanmail.net (J.W.C.); hryun83@gmail.com (H.-R.W.); 2 Department of Otolaryngology-Head and Neck Surgery, Chungnam National University College of Medicine, Daejeon 35015, Korea; jungsn1122@gmail.com (S.-N.J.); dlaaldo22@naver.com (M.A.L.); songkh87@naver.com (K.S.); 3 Division of Endocrinology and Metabolism, Department of Internal Medicine, Chungnam National University College of Medicine, Daejeon 35015, Korea; yeeuni220@naver.com

**Keywords:** head and neck cancer, GDF15, EGR1, progression

## Abstract

Growth and differentiation factor 15 (GDF15), a divergent member of the transforming growth factor-β (TGF-β) superfamily, has been reported to be overexpressed in different kinds of cancer types. However, the function and mechanism of GDF15 in head and neck cancer (HNC) remains unclear. The Cancer Genome Atlas (TCGA) data show that the expression of GDF15 is significantly associated with tumor AJCC stage, lymph vascular invasion and tumor grade in HNC. In this study, we confirmed that knockdown of GDF15 attenuated: cell proliferation, migration and invasion via regulation of EMT through a canonical pathway; SMAD2/3 and noncanonical pathways; PI3K/AKT and MEK/ERK in HNC cell lines. Furthermore, we found that early growth response 1 (EGR1) was a transcription factor of GDF15. Interestingly, we also demonstrated that GDF15 could regulate the expression of EGR1, which meant a positive feedback loop occurred between these two factors. Moreover, combined inhibition of both GDF15 and EGR1 in a HNC mouse xenograft model showed significantly decreased tumor volume compared to inhibition of EGR1 or GDF15 alone. Our study showed that the GDF15–EGR1 signaling axis may be a good target in HNC patients.

## 1. Introduction

Head and neck cancer (HNC) is the sixth most common cancer worldwide. It is typically associated with heavy use of tobacco and alcohol [1,2]. Recently, patients with HPV-associated oropharyngeal cancer have been increasing, predominantly among younger people after oral sex exposure [3]. The standard treatment for patients with HNC includes surgery, chemotherapy, and radiotherapy or combined modality [4]. Although treatment strategies have advanced rapidly, the overall 5-year survival rate for HNC patients has not changed a lot [5]. Therefore, it is absolutely imperative to profoundly understand the biological mechanisms of HNC progression.

Growth and differentiation factor 15 (GDF15) is a distant member of the transforming growth factor-β (TGF-β) family of proteins. Under normal conditions, GDF15 is maintaining in a low level of expression. However, it can be dramatically upregulated under pathological stimulation, such as cardiovascular diseases, endocrine diseases (diabetes and obesity), inflammation and cancer [6,7,8]. The conflicting effect of GDF15 in cancer has not been fully elucidated yet. Some theses indicated that GDF15 had tumor suppressor activity, while other data manifested that it had oncogenic activity [9]. Researchers also uncovered that whether GDF15 is an oncogene or a suppressor gene of tumor growth appears to be depending on the cancer cell type, the stage of the cancer, as well as the location of GDF15 in cancer cells [10,11].

Early growth response 1 (EGR1) is a controversial gene that is involved in growth, differentiation and apoptosis. The protein encoded by this gene belongs to the EGR family of zinc finger proteins [12]. EGR1 is induced by various growth factors, cytokines and stress stimulation, such as radiation and injury [13]. It is a nuclear protein and functions as a multifarious genes’ transcriptional regulator, including tumor suppressor genes and oncogenes. This makes EGR1 a double-edged sword gene in cancer. It was reported that EGR1 was a regulator of various tumor suppressors, including TGF-β, PTEN, p53, and fibronectin, in the fight against cancer [14]. In contrast, it was proposed that EGR1 and TCF7L2 synergistically regulate transcription of LCN2 to promote cell migration in esophageal squamous cell carcinoma [15].

In our study, we clarify that EGR1 is one of the transcription factors of GDF15 in HNC and acts as a protumorigenesis role together with GDF15. In addition, we also demonstrate that GDF15 can regulate the expression of EGR1, which means there is a positive feedback loop between GDF15 and EGR1 in HNC. The highlight of our study is the remarkable effect on suppressing tumor growth after combined inhibition of both GDF15 and EGR1, compared to inhibition of EGR1 or GDF15 alone in HNC mouse models. Our study shows that the GDF15–EGR1 signaling axis may be a good target in HNC patients.

## 2. Results

### 2.1. GDF15 Expression in the Public Database

**Figure 1:**
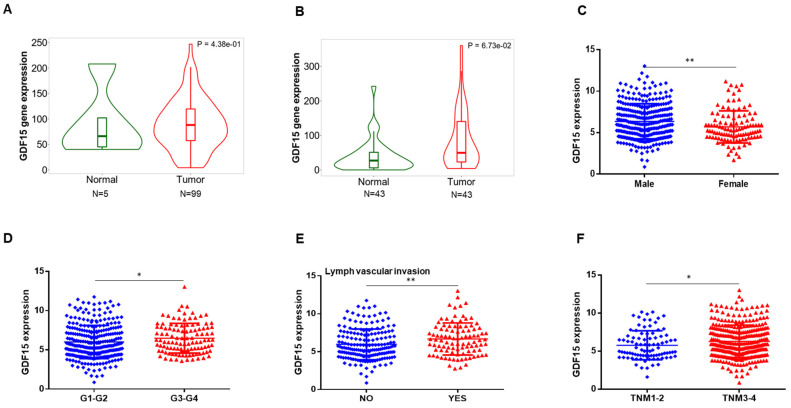
GDF15 expression in HNC patients’ tissues and the correlation analyses between GDF15 expression and clinicopathological characteristics of HNC patients. (**A**,**B**) GDF15 is overexpressed in HNC tissues compared to normal tissues in TNMplot database (left: Genechip, *p* = 4.38 × 10^−1^; right: RNA-seq, *p* = 6.73 × 10^−2^). (**C**–**F**) Expression of GDF15 in HNC based on the Cancer Genome Atlas (TCGA) database against gender, histological grade, lymph vascular invasion and TNM stages. *p* < 0.05 was recognized as statistically significant (* *p* < 0.05, ** *p* < 0.01).

We explored GDF15 expression pattern in the TCGA database. Result shows that the level of GDF15 in tumor tissue tends to be higher than that of normal tissue, although it is not statistically significant (data not shown). In addition, in order to further determine GDF15 mRNA expression in HNC, data from TNMplot were used to support our observations. As a result, GDF15 expression was significantly upregulated both in HNC-Genechip (5 normal head and neck samples versus 99 HNC samples, *p* = 4.38 × 10^−1^) and RNA-seq (43 normal head and neck samples versus 43 HNC samples, *p* = 6.73 × 10^−2^) compared to its expression in the normal tissues (Figure 1A,B). We next used a *t*-test to derive correlations between GDF15 expression and clinicopathological factors affecting the prognoses of HNC patients. As shown in Figure 1C–F, gender (*p* = 0.004), histological grade (*p* = 0.038), lymph vascular invasion (*p* = 0.006) and AJCC stage (*p* = 0.038) are significantly associated with the expression of GDF15. However, there is no difference between the expression of GDF15 with age, T stage and lymph node metastasis (Table S1). Taken together, these data indicated that GDF15 could be playing an oncogenic role in HNC.

### 2.2. GDF15 Promotes Cancer Cell Proliferation, Migration and Invasion

**Figure 2:**
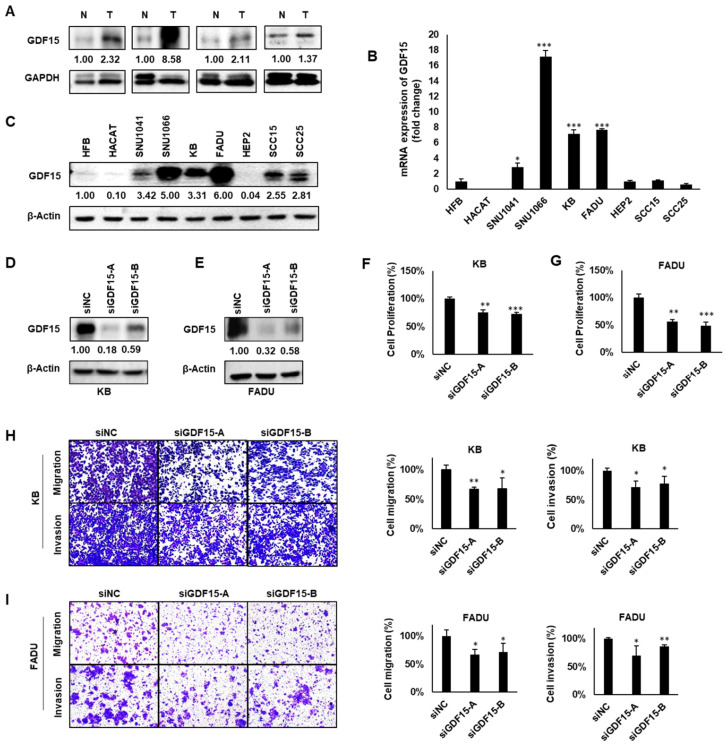
GDF15 expression in HNC. (**A**) Tissue samples obtained from patients with HNC were examined by Western blot analysis using an anti-GDF15 antibody. Representative images of 3 independent experiments are shown. (**B**,**C**) Two normal head and neck cell lines (HFB, HACAT) and seven HNC cell lines (SNU1041, SNU1066, KB, FADU, HEP2, SCC15 and SCC25) were subjected to RT-PCR analysis and Western blot analysis. Representative images of three independent experiments are shown. (**D**–**G**) KB and FADU cells were transiently transfected with GDF15-specific siRNAs or siNC (negative control siRNA) for 48 h. Cell viability was analyzed by the WST-1 assay. The levels of GDF15 were detected by Western blot. (**H**,**I**) After transfection, the cells were allowed to migrate for 24 h in transwell chambers (cell migration) or for 48 h in chambers coated with Matrigel (cell invasion). Magnification, ×40. Differences were considered relevant at *p* < 0.05 (* *p* < 0.05, ** *p* < 0.01, *** *p* < 0.001). All experiments were repeated at least three times.

To explore whether GDF15 plays an oncogenic role in HNC, we evaluated GDF15 expression in normal and tumor tissues derived from the same head and neck cancer patients. Considerably higher expression of GDF15 protein was detected in all of four tumor samples relative to the normal tissues (Figure 2A). The levels of GDF15 mRNA and protein were also examined in two normal cell lines (HFB, HACAT) and in ten head and neck cancer cell lines (SNU1041, SNU1076, KB, SNU1066, FADU, SNU46, SCC15, SCC25, YD8 and HEP2). The majority of HNC cell lines demonstrated notably higher GDF15 expression at both mRNA and protein levels (Figure 2B,C). To investigate the functional significance and mechanism of GDF15 in HNC, we selected two HNC cell lines, KB (exhibiting moderate GDF15 expression) and FADU (exhibiting high GDF15 expression). GDF15-siRNA was used to knock down GDF15 expression in two HNC cell lines (Figure 2D,E). Then, cell proliferation was detected by WST-1 assay. Depletion of GDF15 significantly reduced cell proliferation in KB and FADU cell lines (Figure 2F,G). Cell migration and invasion have been recognized as key steps in tumor metastasis. GDF15 knockdown obviously suppressed the migration and invasion of KB and FADU cells (Figure 2H,I). In contrast, to examine the effect of GDF15 overexpression, HEP2 (exhibiting low GDF15 expression) was used in this experiment. Data showed that overexpressing GDF15 increased cell proliferation, migration and invasion (Figure S1). Together, these data suggest that GDF15 promotes growth and tumorigenicity of HNC cells.

### 2.3. GDF15 Regulates Epithelial–Mesenchymal Transition (EMT)-Related Proteins via Phosphorylating SMAD, ERK and AKT in HNC

**Figure 3:**
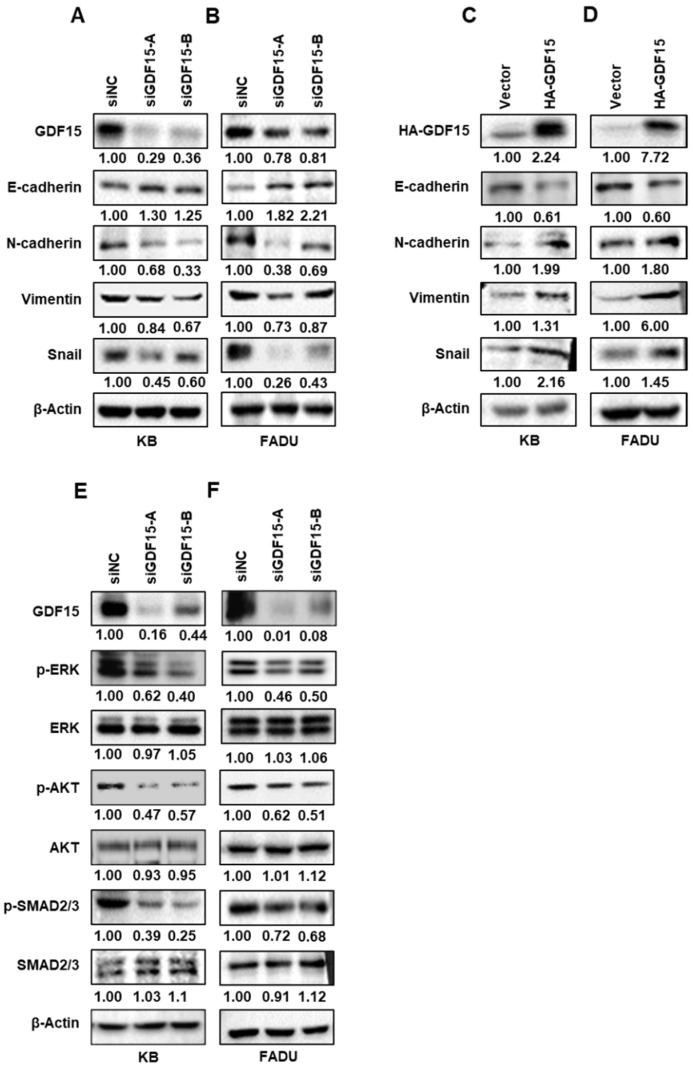
GDF15 promotes EMT-related proteins through SMAD, ERK and AKT signaling pathways in HNC. (**A**–**D**) KB and FADU cells were transiently transfected with GDF15 siRNAs or negative control siRNA for 48 h and HA-GDF15 vector or negative control vector for 24 h. After transfection, the levels of the EMT-related proteins E-cadherin, N-cadherin, vimentin, and Snail were evaluated by Western blots. (**E**,**F**) Representative images of Western blot analysis showing the expression of p-ERK, ERK, p-AKT, AKT, p-SAMD2/3 and SMAD2/3 followed by knockdown of GDF15 in KB and FADU cells. Each figure is representative of three independent experiments.

The association between EMT and cell invasion has been demonstrated in cancer progression. To investigate whether GDF15 promotes EMT, we examined both epithelial and mesenchymal markers, including E-cadherin, N-cadherin, Vimentin and Snail by Western blot analysis. GDF15 knockdown caused a significant decrease in N-cadherin, vimentin and Snail levels and an increase in the level of E-cadherin. On the contrary, overexpression of GDF15 decreased the level of E-cadherin and increased the levels of N-cadherin, vimentin and Snail (Figure 3A–D). The SMAD family axis is a well-known canonical pathway of GDF15. Previous reports also showed that GDF15 plays an important role through the PI3K/AKT and MEK/ERK signaling pathways in various cellular mechanisms [16,17]. We confirmed in our laboratory that knockdown of GDF15 attenuated the expression of p-SMAD2/3, but did not affect total SMAD2/3. In addition, we also demonstrated well-known noncanonical pathways of GDF15, MEK/ERK and PI3K/AKT signaling pathways. We observed that GDF15 suppression significantly decreased AKT and ERK phosphorylation, whereas the total level of AKT and ERK protein was not affected in KB and FADU cells (Figure 3E,F). These results revealed that GDF15 performed its function by modulating EMT via SMAD, PI3K/AKT and MEK/ERK pathways.

### 2.4. EGR1 Is a Transcriptional Regulator of GDF15

**Figure 4:**
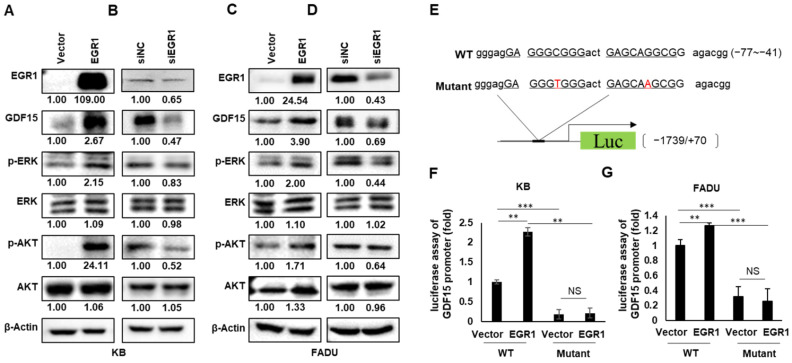
EGR1 induces the expression of GDF15 as its transcription factor. Cells were transfected with an EGR1 overexpression vector (**A**,**C**) or EGR1 siRNA (**B**,**D**), followed by Western blot analysis. After transfection, the levels of GDF15, *p*-ERK, ERK, *p*-AKT and AKT were evaluated by Western blots. (**E**) EGR1 binding region (−77~−41) of GDF15 promoter (−1739/+70) with point mutations (The T and A marked in red) are described. (**F**,**G**) The EGR1 overexpression vector was cotransfected with the wild-type GDF15 promoter or the mutant GDF15 promoter into KB and FADU cells. At 24 h after transfection, cells were lysed and luciferase activity was measured using a luminometer. Differences were considered relevant at *p* < 0.05 (** *p* < 0.01, *** *p* < 0.001). All experiments were repeated at least three times.

We investigated how GDF15 drives HNC progression through the SMAD, PI3K/AKT and MEK/ERK pathways in HNC. To find the upstream factors of GDF15, we searched transcription factor sites (UCSC and TRRUST) and then focused on EGR1 as a potential transcription factor. To determine the association between GDF15 expression and EGR1, we performed an analysis of clinical data in the TCGA database. As shown in Figure S2, we found significant positive correlations between GDF15 and EGR1 (*p* < 0.05). These data further suggest that EGR1 expression is positively associated with GDF15 in HNC. As shown in Figure 4A–D, ectopic expression of EGR1 significantly increased GDF15 protein expression as well as the downstream components of its pathway, *p*-ERK and *p*-AKT, in both KB and FADU cells, but did not change the expression of total AKT and ERK. On the contrary, the expression of GDF15, *p*-ERK, and *p*-AKT significantly decreased after EGR1 knockdown. Next, an EGR1 binding site was found in the GDF15 promoter region from the literature [18]. We identified the EGR1-binding site on our hGDF15 promoter and created a point mutation of the EGR1-binding site on the GDF15 promoter (Figure 4E). To determine whether GDF15 induction was mediated by the direct binding of EGR1 to the promoter of GDF15, we evaluated the effect of EGR1 on the activity of the GDF15 promoter by a luciferase reporting assay. EGR1 overexpression increased the luciferase activity of the wild-type human GDF15 promoter construct. However, EGR1 did not increase the luciferase activity in the mutant form of the human GDF15 promoter in KB cells (Figure 4F). Similar experiments with the FADU cell line yielded analogous results (Figure 4G). Thus, direct binding of EGR1 to the GDF15 promoter plays a critical role in GDF15 expression in HNC cells.

### 2.5. A Positive Feedback Loop of EGR1 and GDF15 Facilitates Proliferation, Migration and Invasion in HNC

**Figure 5:**
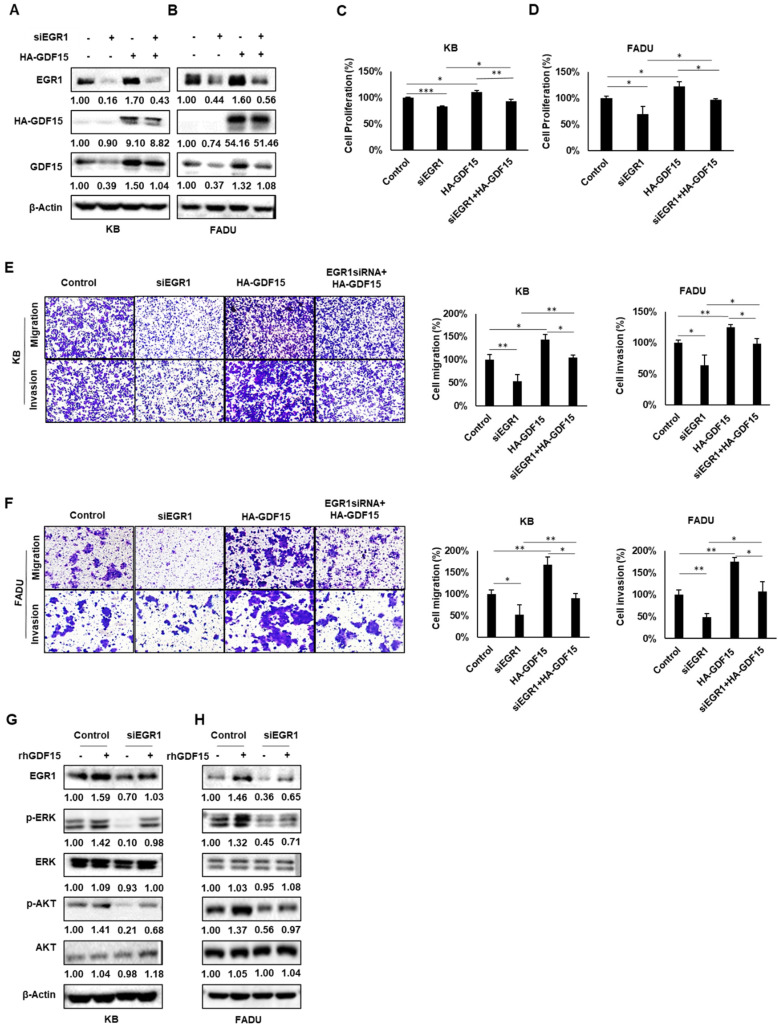
A positive feedback loop between EGR1 and GDF15 facilitates proliferation, migration and invasion in HNC. KB and FADU cells were transiently cotransfected with EGR1 siRNA, the HA-GDF15 overexpression vector or a negative control. After transfection, the expression of GDF15 and EGR1 was evaluated by Western blot analysis (**A**,**B**), and cell viability was evaluated using a WST-1 assay (**C**,**D**). Furthermore, cells were allowed to migrate and invade for 24 h in transwell chambers (cell migration) or for 48 h in chambers coated with Matrigel (cell invasion). Magnification, ×40 (**E**,**F**). (**G**,**H**) KB and FADU cells were transfected with EGR1 siRNA or negative control siRNA for 48 h and then cells were treated with or without rhGDF15 for 2 h (100 ng/mL). Cells were lysed and *p*-ERK, ERK, *p*-AKT, and AKT were detected by Western blots. Differences were considered significant at *p* < 0.05 (* *p* < 0.05, ** *p* < 0.01, *** *p* < 0.001). Each figure is representative of three independent experiments.

To further understand the EGR1–GDF15 signaling pathway, we investigated their interactions. As seen in Figure 5A,B, GDF15 levels decreased after treatment with siEGR1, as expected. On this basis, treatment with HA-GDF15 partially restored the expression of EGR1 in the KB and FADU cell lines. In a functional assay, EGR1 knockdown attenuated the proliferation, migration and invasion of KB and FADU cells, and GDF15 overexpression by treatment with HA-GDF15 after EGR1 knockdown rescued the situation (Figure 5C–F). These results showed that EGR1 is not only an upstream regulator of GDF15 but additionally, GDF15 can affect the expression of EGR1. These data imply that GDF15 can regulate the expression of EGR1 in an autocrine manner.

It has been reported that EGR1 is a downstream component of the MEK/ERK signaling pathway and can promote the cell cycle and invasion of cancer cells through this pathway [19]. Considerable evidence also suggests that GDF15 might contribute to tumor progression through autocrine and paracrine signaling [20,21,22]. Hence, we hypothesized that GDF15 may also regulate EGR1 through phosphorylation of ERK and AKT in a paracrine manner. To investigate this hypothesis, we administered rhGDF15 with or without siEGR1. In Figure 5G,H, we show that EGR1 overexpressed, with increased phosphorylation of ERK and AKT after treatment with rhGDF15. EGR1 knockdown decreased the effect of rhGDF15 on the phosphorylation of ERK and AKT in KB and FADU cells. These data imply that GDF15 can also regulate the expression of EGR1 in a paracrine manner. Altogether, we demonstrated a positive feedback loop between GDF15 and EGR1 in HNC progression.

### 2.6. Combined Treatment Targeting Both GDF15 and EGR1 Synergistically Reduced the Tumor Growth In Vivo

**Figure 6:**
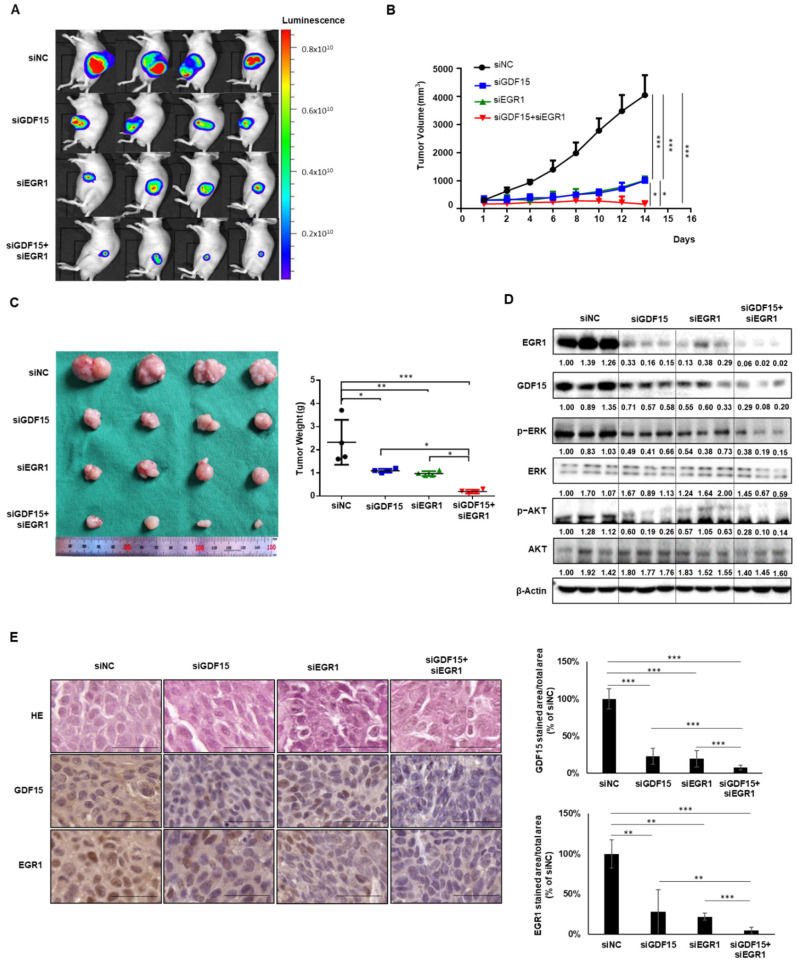

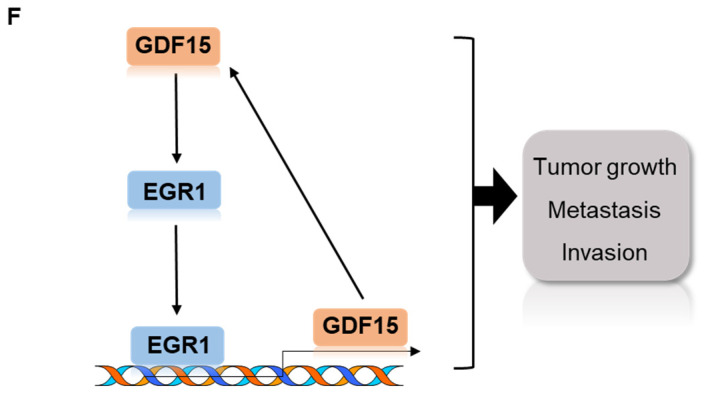
GDF15 can synergistically affect tumor growth of HNC with EGR1 in vivo. Luciferase-expressing FADU (Luc-FADU) HNC cells were injected subcutaneously into BALB/c-nude mice and treated with siGDF15, siEGR1 and cotreated with siGDF15 and siEGR1 or siNC every other day for 2 weeks. (**A**) Final tumor images of cancer cells tracked with the in vivo imaging system following the injection of mice with Luc-FADU cells. (**B**) Tumor growth curve in Luc-FADU-bearing mice that were injected with siGDF15, siEGR1, and cotreated with siGDF15 and siEGR1 or siNC. (**C**) Images of tumors at the experimental endpoint. The tumors were weighed at the time of sacrifice. (**D**) Western blots from xenograft tumor tissues. Changes in the protein expression of GDF15, EGR1, *p*-ERK, ERK, *p*-AKT and AKT in xenograft tissues of the four treatment groups. (**E**) Representative images of HE and immunohistochemical staining of the negative control group, siGDF15 treatment group, siEGR1 treatment group and cotreatment group. Scale bar, 50 μm. Results were analyzed using one-way ANOVA. Data were expressed as mean ± SD. Differences were considered significant at *p* < 0.05 (* *p* < 0.05, ** *p* < 0.01, *** *p* < 0.001). All experiments were repeated two times. (**F**) Schematic diagram of this study. GDF15 promotes cell proliferation and metastasis in HNC through EGR1. Secreted GDF15 can activate SMAD2/3, AKT, ERK, as well as the well-known downstream component of the ERK pathway (EGR1) to form an EGR1-GDF15-ERK-EGR1 positive feedback loop.

To confirm the effects of siGDF15 and siEGR1 treatment on HNC cells in an established xenograft mouse model, bioluminescence imaging was performed to observe changes in tumor cell growth in vivo. The fluorescence area of the siGDF15-treated group and siEGR1-treated group was significantly reduced compared to the control group. In addition, the cotreatment group had a significantly smaller fluorescence area than the groups treated with siGDF15 or siEGR1 alone (Figure 6A). After sacrificing the mice, tumor volume and weight were measured immediately. The tumor volume and weight were significantly lower in the cotreatment group than in the single treatment groups (Figure 6B,C). We confirmed that the protein expression of *p*-ERK and *p*-AKT in tumor tissues synergistically decreased along with GDF15 and EGR1 in the combined treatment group compared to the single treatment groups, whereas the total level of AKT and ERK protein were not affected (Figure 6D). Immunohistochemistry also showed lower levels of both GDF15 and EGR1 in the cotreatment group than in the single treatment groups (Figure 6E). These results suggest that knockdown of both GDF15 and EGR1 has a clear advantage over knockdown of GDF15 or EGR1 alone in terms of an inhibitory effect against tumor growth in the in vivo model. Our findings show that the GDF15–EGR1 signaling axis may play a pivotal role in the regulation of tumor progression, since GDF15 activated tumor progression via EGR1 activation and EGR1 positively regulated GDF15 expression in HNC cells (Figure 6F).

## 3. Discussion

Despite recent advances in treatment technology, the survival rate for HNC patients remains roughly 50% [23]. The carcinogenesis of HNC involves numerous molecular events such as the sequential activation of oncogenes and the inactivation of tumor suppressor genes [24]. Although epidermal growth factor receptor (EGFR) inhibitors have been approved as targeted agents for HNC, they are not effective for everyone, and once resistance develops, there are few suitable alternative target drugs for HNC patients [25]. Therefore, the need for additional treatment options that improve HNC outcomes is pressing.

In this study, we verified that the expression of GDF15 in HNC tumor tissues and cell lines was elevated, and its overexpression was closely related with the AJCC stage, lymphovascular invasion, and tumor grade. Overexpression of GDF15 also promoted proliferation, migration and invasion of HNC cells. Our results are consistent with the findings of previous studies [26,27]. Large-scale analyses of biomarkers from cancer samples showed elevated expression of GDF15 in the tissue or serum of patients with prostate, breast and colorectal carcinomas [28]. GDF15 was also reported to be highly expressed in multiple myeloma, malignant melanoma, ovarian cancer and gastric cancer [29]. Based on the expression patterns of GDF15 reported in various cancer types and our data, GDF15 could be considered a potential prognostic biomarker to improve the risk assessment for cancer progression in HNC [27,30].

It has been reported that GDF15 is involved in cancer progression through noncanonical pathways (PI3K/AKT and MEK/ERK) [21,31,32,33,34], as well as the canonical TGF-β/SMAD signaling pathway [35,36]. In the present study, we also suggested that GDF15 regulates EMT-related proteins via the activation of both noncanonical (PI3K/AKT and MEK/ERK) and canonical (SMAD2/3) signaling pathways. We then sought to identify the upstream regulator of GDF15 in HNC. We focused on EGR1 as a transcription factor of GDF15 that belongs to the early growth response family. Previous studies showed that EGR1 regulates the transactivation of genes involved in tumor growth inhibition as an anticancer gene in cancer, and that EGR1 inhibits proliferation, migration and invasion [37,38]. Other studies also reported that GDF15 contributes to cancer cell apoptosis due to the upregulation of EGR1 as a critical antitumorigenesis role [18,39,40,41,42]. Furthermore, another study also showed that EGR1 acts as a key regulator of prostate cancer through the suppression of GDF15 [43]. In contrast, some studies showed that EGR1 was involved downstream of the MEK/ERK-JNK signaling pathway, with protumorigenic effects. The activated MAPK pathway enhanced the interaction of EGR1 and cyclin D1, and then increased the cyclin D1 protein level in prostate cancer cells [44]. It was also reported that capecitabine and lapatinib, which inhibit both EGR1 and EGR2, had similar overall survival results to those achieved with the standard treatment of cisplatin, fluorouracil and cetuximab, but with fewer toxic effects [45]. It was reported that EGR1 expression was required for the osteocyte-derived GDF15-mediated induction of in vitro prostate cancer cell proliferation, migration and invasion [46]. In our study, we demonstrated that EGR1 regulates the transcriptional level of GDF15 in HNC cell progression. Notably, we also found that GDF15 overexpression promoted proliferation, migration and invasion via EGR1 upregulation. These findings mean that there is a positive feedback loop between GDF15 and EGR1. Combined treatment with both GDF15 and EGR1 knockdown resulted in significantly reduced tumor formation, suggesting that the GDF15–EGR1 signaling pathway plays a critical role in HNC progression. Although a previous study on bone metastasis of prostate cancer also showed that GDF15 in osteocytes promoted EGR1 expression in prostate cancer cells and that enhanced EGR1 promoted the growth and invasive of prostate cancer cells [46], there are no previous reports of a GDF15–EGR1 positive feedback loop in tumor progression. We showed for the first time that the GDF15–EGR1 signaling axis can synergistically accelerate HNC progression.

However, this study has limitations in that we did not fully investigate how MAPK/ERK and AKT signaling, the main signaling pathway of HNC, is linked to oncogenic capacity of GDF15–EGR1 signaling.

In HNC, elevated GDF15 promotes cancer progression via transcriptional regulation by EGR1. GDF15 also regulates the expression of EGR1. Moreover, combined inhibition of both GDF15 and EGR1 showed significantly decreased tumor volume compared to inhibition of EGR1 or GDF15 alone. Therefore, our study showed that the GDF15–EGR1 signaling axis may be one of the targets for HNC patients.

## 4. Materials and Methods

### 4.1. Cell Lines and Materials

The normal human cell lines HFB and HACAT, and the head and neck cancer cell lines SNU1041, SNU1076, KB, SNU1066, FADU, SNU46, SCC15, SCC25, YD8 and HEP2 were obtained from the KCLB (Korean Cell Line Bank, Seoul, Korea). HFB, HACAT, KB, and FADU cells were maintained in high-glucose DMEM (Gibco, Grand Island, NY, USA). SNU1041, SNU1076, SNU1066, SNU46 and YD8 were cultured in RPMI-1640 (Welgene, Gyeongsan, Korea), and SCC-15 and SCC25 were cultured in DMEM/F12 (Welgene). HEP2 cells were cultured in EMEM (ATCC, Manassas, VA, USA). All cells were supplemented with 10% fetal bovine serum (FBS) and 5% penicillin-streptomycin (Gibco). Cells were grown at 37 °C with 5% CO_2_ under humidified conditions.

### 4.2. RNA Isolation and Reverse Transcription-Polymerase Chain Reaction (RT-PCR)

Total cellular RNA was extracted using TRIzol (Invitrogen, Carlsbad, CA, USA) and cDNA was synthesized with 2 µg total RNA and TOPscriptTMRT DryMIX (Enzynomics Inc., Daejeon, Korea) according to the manufacturer’s instructions. Amplification was carried out using SYBR Green qPCR master mix (Thermo Fisher Scientific, Waltham, MA, USA). The PCR reactions were performed for 40 cycles at 95 °C for 15 s, 60 °C for 1 min and 72 °C for 1 min. The primer sequences were as follows: GDF15-F: 5′-TCA GAT GCT CCT GGT GTT GC-3′/GDF15-R: GAT CCC GAA AGC CGC ACT TCT G-3′; GAPDH-F: 5′-ACC CAG AAG ACT GTG GAT GG-3′/GAPDH-R: 5′-TTC TAG ACG GCA GGT CAG GT-3′. The Ct values provided from real-time PCR instrumentation were imported into Microsoft Excel. We used the 2^−∆∆Ct^ model for relative quantification of real-time qPCR fold changes [47].

### 4.3. Western Blot Analysis

Cells were lysed in a buffer containing 150 mM NaCl, 1.0% nonidet-P40, 0.5% sodium deoxycholate, 0.1% sodium dodecyl sulfate, 50 mM Tris (pH 8.0) and a protease inhibitor cocktail (Roche Applied Science, Vienna, Austria, pH 7.4). Frozen tissue samples stored in liquid nitrogen were minced with scissors. Each sample was homogenized in a lysis buffer at a ratio of 1:20 *w*/*v*. After centrifugation at 13,000 rpm for 20 min, the supernatant was used to measure the total protein. Electrophoresis was performed as described previously [48]. The following primary antibodies were used for Western blot analysis: anti-GDF15 (1:1000; Abcam, Cambridge, UK), anti-phospho-AKT (Ser 473), anti-total AKT, anti-phospho-ERK, anti-total ERK, anti-phospho-SMAD2/3, anti-total SMAD2/3, anti-EGR1, anti-vimentin, anti-Snail, anti-β-actin (1:1000; Cell Signaling Technology Inc., Danvers, MA, USA), anti-N-cadherin, anti-E-cadherin and anti-GAPDH (1:1000; Santa Cruz Biotechnology, Dallas, TX, USA). Following incubation with the corresponding horseradish peroxidase-conjugated secondary antibodies (1:5000; Santa Cruz Biotechnology), immune reactive bands were visualized by enhanced chemiluminescence detection (Bio-Rad Laboratories, Inc., Hercules, CA, USA).

### 4.4. Small-Interfering RNA (siRNA) Transfection

Transient transfection was performed once cells reached 60% confluence using Lipofectamine RNAiMAX reagent (Invitrogen) for siRNA and jetPEI DNA transfection reagent (Polyplus, Illkirch-Graffenstaden, France) for the overexpression vector, following the manufacturers’ standard protocols. The siRNA for GDF15 was acquired from Invitrogen, the siRNA for EGR1 was acquired from Santa Cruz Biotechnology and the siRNA for the control group was acquired from Bioneer (Daejeon, Korea). The SAMiRNA for GDF15, EGR1 and control for in vivo experiments was acquired from Bioneer. The overexpression vector for HA-GDF15 and EGR1 was obtained from OriGene Technologies (Rockville, MD, USA). All experiments were repeated at least three times.

### 4.5. Cell Proliferation Assay

Cells were seeded at a density of 5 × 10^3^ cells per well in 96-well plates in DMEM containing 10% FBS. After transfection with siRNA for 48 h or with the overexpression vector for 24 h, cell viability was measured using the cell proliferation reagent WST-1 (Roche Diagnostics, Indianapolis, IN, USA). WST-1 formazan was quantitated at 450 nm using an enzyme-linked immunosorbent assay reader. Results are presented as percentages relative to control cells.

### 4.6. Cell Migration and Invasion Assay

Transwell membranes (24-well; Costar, Cambridge, MA, USA) were coated with Matrigel for 6 h for the invasion assay or without Matrigel for the migration assay. In total, 2 × 10^5^ cells in serum-free medium were seeded onto the upper chamber, and 750 μL of medium with 10% FBS was added to the lower chamber. After incubation for 24 h (for migration) and 48 h (for invasion), the cells adhering to the upper surface of the membrane were removed with a cotton swab. The invasion or migration cells, which adhered to the lower surface, were stained with crystal violet and counted in 4 representative fields by light microscopy (×40 magnification).

### 4.7. Plasmid Constructs and Site-Directed Mutagenesis

The pGL3B-human GDF15 (−1739/+70) luciferase reporter constructs were provided by Dr. Y. Moon (Pusan National University, Pusan, Korea). We found the EGR1-binding site (gggag GA GGGCGGG act GAGCAGGCGG agacgg: the uppercase letters represent the binding parts) of the GDF15 promoter according to previous research [18]. To create an EGR1 point-mutant reporter (GDF15 EGR1 mut-Luc [1739/+70]), the 5′-gggag GA GGGCGGG act GAGCAGGCGG agacgg-3′ sequence of the promoter binding site was mutated to 5′-gggag GA GGGTGGG act GAGCAAGCGG agacgg-3′ using DpnI-based site-directed mutagenesis (Agilent Technologies, Santa Clara, CA, USA). The nucleotide sequences of all plasmids were confirmed by automated sequencing.

### 4.8. Luciferase Assay

KB and FADU cells were plated in 6-well culture plates and cotransfected with the EGR1 overexpression vector or empty pCMV6-Entry with the human GDF15 (−1739/+70) or mutant form of human GDF15 (−1739/+70) luciferase reporter using the jetPEI reagent. At 24 h post-transfection, cells were lysed and luciferase assays were performed using a luciferase assay kit (Promega, Fitchburg, WI, USA) following the manufacturer’s instructions. All assays were performed at least in triplicate.

### 4.9. Animal Experiments

Six-week-old BALB/c nude mice were obtained from Orient Bio (Seongnam, Korea). The animals were housed at 24 °C with a 12 h day/night cycle under specific pathogen-free conditions. They had ad libitum access to a gamma-ray-irradiated laboratory rodent diet (Purina Korea) and autoclaved water. All experiments were performed in accordance with the relevant guidelines and regulations of the animal care unit at Chungnam National University. The animal protocols for these experiments were approved by the Ethics Committee of Animal Experimentation of Chungnam National University (No. CNUH-020-A0036-1). Luciferase-expressing FADU (Luc-FADU) cells were subcutaneously inoculated into the lower left flanks of BALB/c nude mice. The mice were randomly divided into four groups: negative control siRNA (siNC), GDF15-siRNA (siGDF15), EGR1-siRNA (siEGR1), and cotransfected with siGDF15 and siEGR1. Two weeks later, when the tumors reached ~5 mm in diameter, the tumors were injected with siNC, siGDF15, siEGR1, or cotreatment with siGDF15 and siEGR1 every other day. Body weight was recorded periodically. Tumor dimensions were measured using a caliper, and tumor volumes were estimated as follows: tumor volume = length × width^2^ × 0.52, where length represents the largest tumor diameter and width represents the diameter perpendicular to the length. At the experimental endpoint, the tumors were harvested and used for histological analyses. All of the animal experiments were repeated at least twice with similar results.

### 4.10. In Vivo Imaging

Bioluminescence imaging was performed using an in vivo imaging system consisting of a Lumina XRMS instrument (PerkinElmer, Waltham, MA, USA). To obtain in vivo bioluminescence imaging, animals were intraperitoneally administered 150 mg/kg D-luciferin (Promega). Images were acquired and analyzed using the Living Image software program (Caliper Life Sciences, Waltham, MA, USA). After anesthetizing the mice with 2% isoflurane in 100% O_2_, bioluminescence images were taken.

### 4.11. Histological and Immunohistochemical Analysis

Tissue samples were fixed in 4% formalin solution and paraffin embedded. For hematoxylin and eosin (HE) staining, tissue sections were deparaffinized in xylene, hydrated in graded alcohol solutions and stained with HE. The samples were examined under an automatic digital slide scanner (Pannoramic MIDI) after mounting. For immunohistochemistry, tissue sections were deparaffinized in xylene, hydrated in graded alcohol solutions and heated (100 °C) for 15 min in Antigen Retrieval Citra Solution (pH 6.0) for antigen retrieval. For single immunostaining, endogenous peroxidase activity was blocked in a 1% hydrogen peroxide solution (Sigma-Aldrich, St. Louis, MO, USA) in PBS with 0.3% Triton X-100 for 30 min at room temperature. The sections were incubated with the indicated antibodies overnight at 4 °C and then incubated with the corresponding horseradish peroxidase-conjugated secondary antibody. Finally, 3,3′-diaminobenzidine (DAB; Dako, Agilent) was used to detect these labeled antibodies and the nucleus was stained with hematoxylin. After rinsing with PBS, the samples were mounted and analyzed using an automatic digital slide scanner (Pannoramic MIDI).

### 4.12. Statistical Analysis

All in vitro experiments were repeated three times and statistical significance was analyzed using Student’s *t*-test. The in vivo results were analyzed using a one-way ANOVA. Data are presented as means ± standard deviation (SD). To evaluate associations between GDF15 and EGR1 expression in The Cancer Genome Atlas (TCGA) data, we used Pearson correlation analyses. A *p*-value < 0.05 was considered to indicate statistical significance. All statistical analyses were performed using SPSS version 26.0 (IBM Corp., Armonk, NY, USA).

## Data Availability

The data presented in this study are openly available in the Cancer Genome Atlas (TCGA) database (www.cancer.gov/about-nci/organization/ccg/research/structural-genomics/tcga/using-tcga, accessed on 19 September 2021) and University of California Santa Cruz (UCSC) database (genome.ucsc.edu).

## Supplementary Materials

The following are available online at https://www.mdpi.com/article/10.3390/ijms222011151/s1.

## Abbreviations

GDF15	Growth and differentiation factor 15
EGR1	Early growth response 1
TGF-β1	Transforming growth factor β1
p53	Tumor protein p53
HE	Hematoxylin and eosin
RT-PCR	Quantitative real-time reverse transcriptase polymerase chain reaction
cDNA	Complementary DNA
GAPDH	Glyceraldehyde-3-phosphate dehydrogenase
