# Supplementary material for: Transcriptional Regulation of GDF15 by EGR1 Promotes Head and Neck Cancer Progression through a Positive Feedback Loop
Source: Int J Mol Sci. 2021 Oct 15;22(20):11151. doi: 10.3390/ijms222011151 (PMC8538541; doi:10.3390/ijms222011151)
Supplement: Supplementary file 1 [file ijms-22-11151-s001.zip › Revised supplementary figure-YANLI JIN docx.pdf]

International Journal of *Molecular Sciences*

MDPI

Supplementary data

Transcriptional Regulation of GDF15 by EGR1 Promotes Head and Neck Cancer Progression through a Positive Feedback Loop

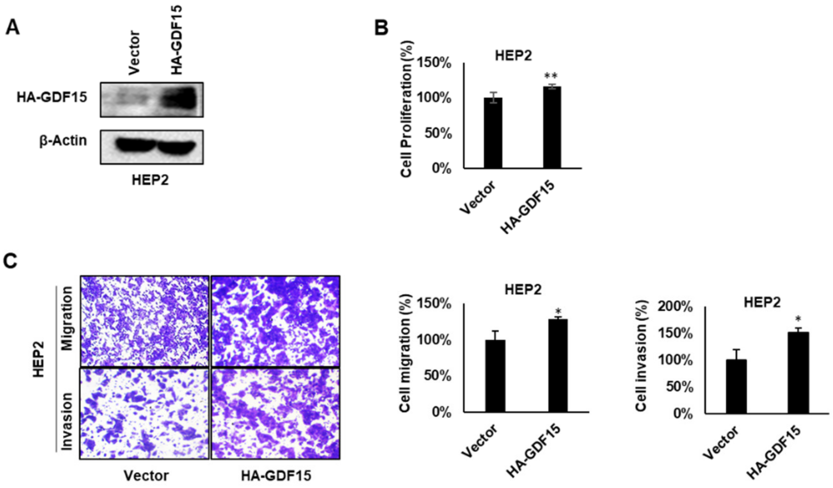

Figure S1. Overexpression of GDF15 increases proliferation, migration, and invasion of HEP2 cells. HEP2 cells were transiently transfected with HA-GDF15 overexpression vector or negative control for 24 h. After transfection, the expression of HA-GDF15 were evaluated by western blot analysis (A). Cell viability was then evaluated using the WST-1 assay (B). (C) After transfection, the cells were allowed to migrate for 24 h in Transwell chambers (cell migration) or for 48 hours in chambers coated with Matrigel (cell invasion). All experiments were repeated at least three times. Differences were considered significant at $p<0.05$ (* $p<0.05$, ** $p<0.01$).

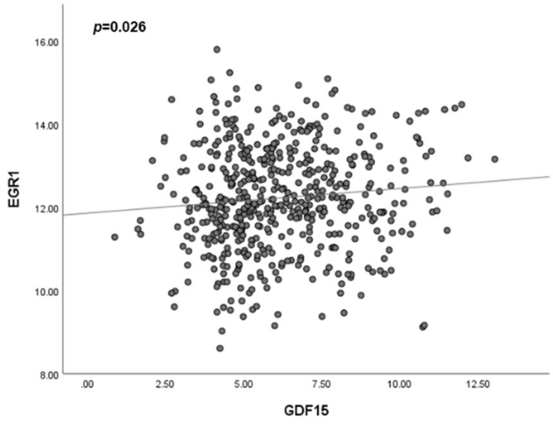

Figure S2. Pearson correlation analysis confirmed that there was a statistically significant correlation between GDF15 and EGR1. This result was obtained from TCGA HNC data.
