# Supplementary material for: Transcriptional Regulation of GDF15 by EGR1 Promotes Head and Neck Cancer Progression through a Positive Feedback Loop
Source: Int J Mol Sci. 2021 Oct 15;22(20):11151. doi: 10.3390/ijms222011151 (PMC8538541; doi:10.3390/ijms222011151)
Supplement: Supplementary file 1 [file ijms-22-11151-s001.zip › Revised supplementary table-YANLI JINdocx.pdf]

International Journal of
Molecular Sciences

MDPI

Supplementary table

Transcriptional Regulation of GDF15 by EGR1 Promotes Head and Neck Cancer Progression through a Positive Feedback Loop

Table S1. The correlation of GDF15 expression with the clinical pathological characteristics of TCGA-HNC cohort.

Characteristics	No. of patients	GDF15 expression	
		Mean ± SD	*p*-value
**Age, years**			
≤ 65	277	6.26±2.05	
> 65	144	6.06±2.15	0.363
**Gender**			
Male	312	6.36±2.12	
Female	109	5.69±1.93	0.004*
**Lymph vascular invasion**			
No	189	5.94±2.01	
Yes	98	6.68±2.12	0.004*
**Lymph node metastasis**			
No	197	6.08±2.18	
Yes	217	6.33±2.02	0.232
**Neoplasm disease stage**			
G1-G2	298	6.01±2.11	
G3-G4	111	6.48±1.89	0.038*
**T stage**			
T1-T2	156	6.18±2.19	
T3-T4	264	6.20±2.03	0.903
**TNM stage**			
I + II	91	5.79±1.91	
III + IV	330	6.30±2.13	0.038*
**Alcohol consumption**			
No	126	6.00±2.23	
Yes	286	6.25±2.04	0.279
**Survival**			
Yes	269	6.18±2.16	
No	154	6.19±1.97	0.959

TCGA-HNC, The Cancer Genome Atlas Head and Neck Cancer. * $p < 0.05$.
